# Genetic associations between Rapid Eye Movement (REM) sleep behavior disorder and cardiovascular diseases

**DOI:** 10.1371/journal.pone.0301112

**Published:** 2024-05-21

**Authors:** Pengfei Xu, Yitong Wei, Haibo Wu, Li Zhang

**Affiliations:** 1 Department of Neurosurgery, Nanyang Central Hospital, Nanyang, Henan, China; 2 Department of Neurology, Nanyang Central Hospital, Nanyang, Henan, China; Brigham and Women’s Hospital and Harvard Medical School, UNITED STATES

## Abstract

**Background:**

Previous studies revealed that sleep disorders are potential risk factors for cardiovascular diseases, such as obstructive sleep apnea and rapid eye movement (REM) sleep behavior disorder (RBD). However, the causal associations between RBD and cardiovascular diseases remained unknown.

**Materials and methods:**

We used the latest and largest summary-level genome-wide association studies of RBD, stroke and its subtypes, coronary artery disease (CAD), myocardial infarction (MI), and heart failure (HF) to select genetic variants as the instrumental variables. Mendelian randomization (MR) analysis was performed to test the causal associations between RBD and the cardiovascular diseases above. Inverse variance weighted method was used as the main analysis.

**Results:**

After multiple comparisons, genetically predicted RBD was significantly associated with the risk of HF [odds ratio (OR) = 1.033, 95% CI 1.013–1.052, *p* = 0.001]. Leave-one-out analysis further supported the robustness of the causal association. Furthermore, we identified a suggestive association between genetically predicted MI and RBD (OR = 0.716, 95% CI 0.546–0.940, *p* = 0.016). However, in our study no associations were identified of RBD with CAD or stroke and its subtypes.

**Conclusion:**

Our study highlighted the potential associations between RBD and cardiovascular diseases at genetic level, including HF and MI. More studies were required to clarify the biological mechanisms involved the associations.

## Introduction

Cardiovascular diseases, including coronary artery disease (CAD) and stroke are the leading cause of disability and mortality worldwide, and it is estimated that there are nearly 10 million new CAD and stroke cases each year around the globe [1, 2]. The primary prevention for cardiovascular diseases and the early identification of those at a high risk of developing cardiovascular diseases are of great importance [1, 3, 4]. Enormous evidence have proposed the modifiable risk factors for cardiovascular diseases, including hypertension, diabetes mellitus, dyslipidemia, history of smoking, and obesity [5]. And previous clinical trials have also demonstrated that the control of blood pressure, glycemic traits, and blood lipids would significantly reduce the risk of cardiovascular diseases and their recurrence [5]. In addition, recent studies have reported that sleep duration and sleep-related disorder were underlying risk factors for cardiovascular diseases. Wang et al. demonstrated that either shorted or longer sleep duration was associated with the risk of CAD [6]. And a healthy sleep pattern characterized by early chronotype, sleep 7–8 hours per day, rarely insomnia, no snoring, or no excessive daytime sleepiness was associated with a lower risk of heart failure (HF) [7]. For stroke, in a cohort with 79,881 individuals, Titova et al. reported an increased risk of stroke due to longer sleep duration [8]. In addition, sleep-disordered breathing, particularly sleep apnea, was a risk factor for the incidence, recurrence, and poor prognosis of ischemic stroke (IS) [9].

Rapid eye movement (REM) sleep behavior disorder (RBD) is a common neurological disorder defined as the loss of muscle atonia and dream enactment during REM sleep and is a predictive clinical marker for neurodegenerative disorders including Parkinson’s disease, dementia with Lewy body, and multiple system atrophy [10, 11]. However, the association between RBD and the risk of cardiovascular diseases was rarely reported. In a community-based study with more than 10,000 participants, probable RBD was associated with a higher risk of both ischemic and hemorrhagic stroke during a 3-year follow-up [12]. Pace et al. observed significant decrease in REM sleep in patients with acute IS, which was further associated with the poorer functional outcomes of IS [13]. While the associations between RBD and other cardiovascular diseases, including CAD, myocardial infarction (MI), or HF were not reported. In addition, the results of observational research may be biased by small sample sizes, reverse causalities, and residual confounding factors. The gold standard for establishing a causal association is random clinical trial (RCT), which costs much money and takes a lot of time.

**Fig 1:**
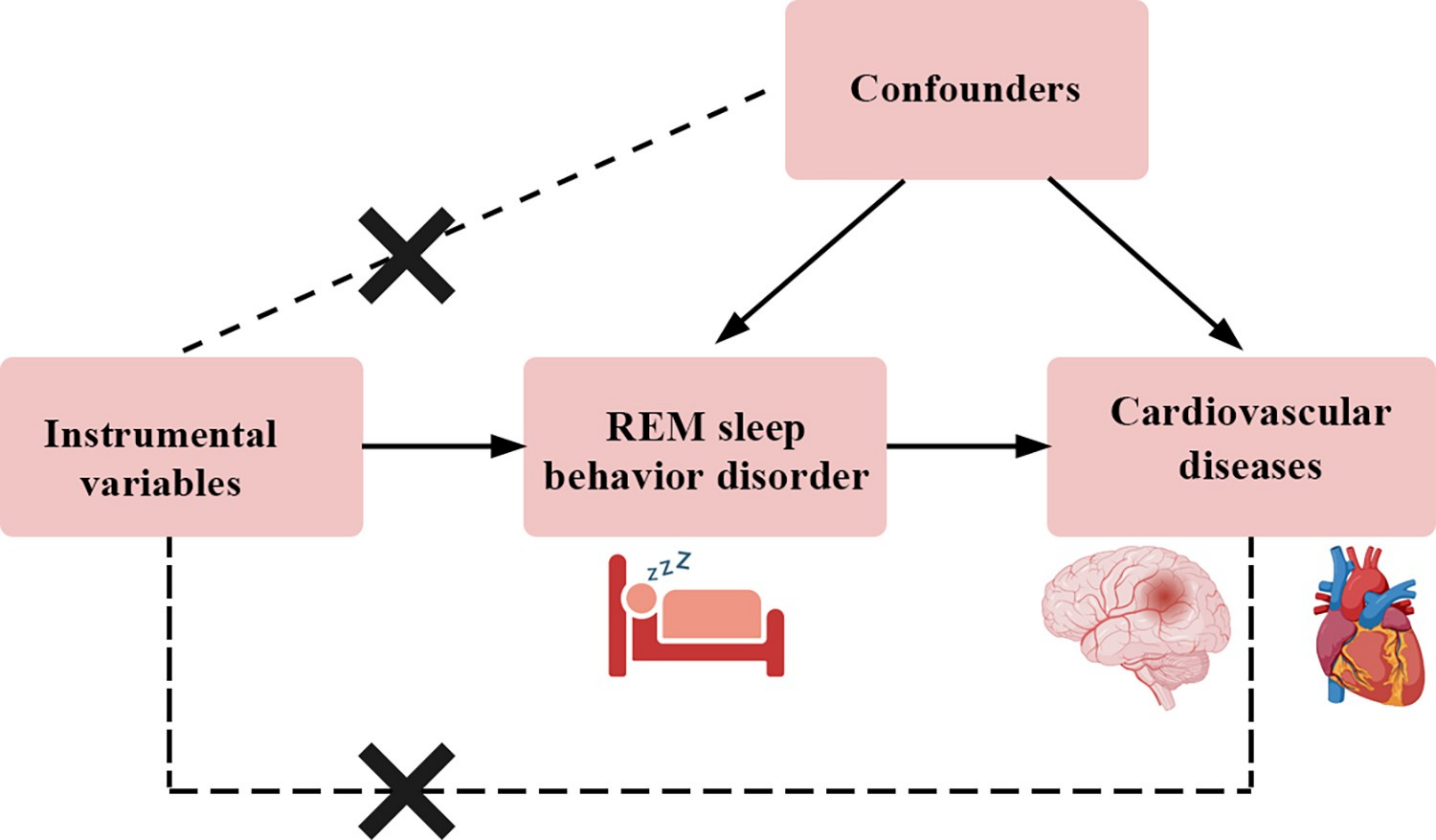
The major assumptions for the Mendelian randomization analysis between REM sleep behavior disorder and cardiovascular diseases. REM: rapid eye movement.

Mendelian randomization (MR) analysis is a genetic approach for inferring causal associations between the exposures and the outcomes [14, 15]. MR analysis uses genetic variants (single nucleotide polymorphisms [SNPs]) as instrumental variables (IVs) [14, 15]. And MR study was based on three major assumptions. First, the genetic variants are strongly associated with the exposures; second, the genetic variants are not associated with confounders; third, the genetic variants are associated with the outcomes through the exposures, but not through alternative pathways or confounders [14, 15]. As the genetic variants were randomly allocated, MR analysis can reduce the effect of reverse causation [14, 15]. In addition, the genome-wide association study (GWAS) datasets used in MR analysis consists of a lot of individuals and the instrumental variables are strongly associated with the exposures, which will reduce the influence of confounding factors [14, 15]. Recent large-scale GWASs have identified multiple genetic loci associated with the risk of RBD [16], so it is possible to analyze the causal associations between RBD and cardiovascular diseases. Therefore, the aim of this study is to test the causal associations between RBD and cardiovascular diseases, including stroke and its subtypes, CAD, MI, and HF through MR approach (**Fig 1**). In addition, we also performed a bidirectional MR analysis to test whether cardiovascular diseases would increase the risk of RBD or not.

## Methods

### RBD GWAS

We obtained the summary-level GWAS dataset for RBD from a recent GWAS meta-analysis by Krohn et al. with 1,061 cases with isolated RBD and 8,386 cases [16]. The GWAS was performed using logistic regression model, adjusting for age, gender, genotype platform, and the top 5 principal components [16]. The participants in the RBD GWAS were all of European ancestry.

### Cardiovascular diseases GWAS

Regarding stroke, we obtained the summary-level GWAS dataset from a GWAS meta-analysis by MEGASTROKE consortium with 40,585 any stroke (AS) cases and 406,111 controls [17]. The AS cases included 34,217 any IS (AIS) cases and the AIS cases could be further subclassified based on the etiological subtypes of IS, including large artery atherosclerosis stroke (LAA, *n* = 4,373), cardioembolic stroke (CES, *n* = 7,193), small artery occlusion stroke (SAO, *n* = 5,386) [17].

Regarding other cardiovascular diseases, we obtained the summary-level CAD GWAS dataset from UK Biobank and CARDIoGRAMplusC4D with 122,733 CAD cases and 424,528 controls [18]. We obtained summary-level MI GWAS dataset by Hartiala et al. with 14,825 MI cases and 44,000 controls [19]. We obtained the summary-level HF GWAS dataset from HERMES consortium with 47,309 HF cases and 930,014 controls [20]. We have confirmed that the participants in the cardiovascular diseases GWAS datasets were all of European ancestry and there was no sample overlap between the RBD GWAS and the cardiovascular diseases GWAS.

### Selection of instrumental variables

To select the instrumental variables for the exposure, we first screened the SNPs significantly associated with the exposure (*p* < 1 × 10^−6^ for RBD and *p* < 5 × 10^−8^ for cardiovascular diseases, respectively). The SNPs with low minor allele frequency (MAF < 0.01) were removed and then the linkage disequilibrium of the SNPs was tested with the reference from the 1000 Genomes Project in European ancestry [21]. The SNPs with the *r*^2^ < 0.001 at a distance of 10,000 kilo base pairs were also excluded. Next we calculated the variance explained (*R*^2^) by each SNP with the formula: *R*^2^ = 2 × *β*^2^ × MAF × (1-MAF), where *β* represented the effect of the SNP on the exposure [22], and the *F*-statistic of each SNP with the formula: *F*-statistic = *R*^2^(*N*-2)/(1- *R*^2^), *N* indicated the sample size [22]. The SNPs with *F*-statistic > 10 remained and were further included in the MR analysis [22]. If the SNP was not identified in the outcome GWAS dataset, a proxy SNP with *r*^2^ > 0.8 was found and substituted.

### MR analysis

Mendelian randomization analysis was conducted in the R software (version 4.0.3) using the R package TwoSampleMR [23]. Inverse variance weighted (IVW), weighted median, weighted mode, simple mode, and MR Egger regression methods were performed in MR analysis [24–26]. The IVW method, which could combine the estimated effects of the SNPs to provide an unbiased estimated effect in the absence of heterogeneities or pleiotropies, was used as the main analysis [27]. Regarding heterogeneity analysis, Cochran’s Q statistic was calculated. And a random effect model of IVW method was conducted if the *p*-value for Q-statistic was less than 0.05. The intercept test was performed by MR Egger regression to test pleiotropy. In addition, leave-one-out analysis was performed to see whether the results of MR analysis were robust when excluding any single SNP. Bonferroni correction was used for multiple comparisons (adjusted *p*-value = 0.05/8 = 0.006). The online analytic tool mRnd (https://cnsge.nomics.shiny.apps.IO/MRND/) was used to estimate the power of MR analysis.

### Ethic approval

This study was based on the publicly available summary-level GWAS data from the studies that had obtained ethical approval and no ethical approval was required for our study.

## Results

### Instrumental variables selection for RBD

There were 12 SNPs selected as instrumental variables for RBD and we have confirmed that all SNPs were strong instruments (all *F*-statistic > 10; **Table 1**). The 12 SNPs explained 39.9% of the variance (**Table 1**). The estimated effect of each single SNP on each cardiovascular disease was shown in **S1 Table**.

**Table 1:** Instrumental variables for rapid eye movement sleep behavior disorder.

SNP	CHR	BP	A1	A2	EAF	BETA	SE	P	*R* ^2^	*F*
rs274759	1	36572161	T	G	0.14	-0.432	0.087	6.41E-07	4.40%	434.26
rs148224267	1	214460766	G	C	0.19	-0.332	0.067	8.65E-07	3.44%	336.09
rs360284	2	127076595	A	G	0.05	0.693	0.139	6.38E-07	4.52%	446.76
rs13022991	2	229998621	A	G	0.13	0.334	0.068	8.23E-07	2.51%	243.33
rs3756059	4	90757272	A	G	0.50	0.401	0.049	2.42E-16	8.03%	824.79
rs140857507	5	154034581	A	G	0.01	1.025	0.206	6.40E-07	2.19%	211.04
rs76917400	6	136144488	G	A	0.08	0.441	0.086	2.94E-07	2.90%	281.81
rs71456122	11	63296797	A	G	0.02	0.901	0.180	5.40E-07	2.70%	261.86
rs73157595	12	101235170	C	T	0.02	0.767	0.154	6.36E-07	2.13%	205.70
rs142735667	14	38717515	A	G	0.01	0.947	0.188	5.10E-07	1.95%	188.01
rs11622216	14	103051901	C	A	0.29	0.255	0.052	7.76E-07	2.70%	262.13
rs16947905	16	49761670	A	G	0.05	0.491	0.092	8.36E-08	2.44%	236.54

A1: effect allele; A2: other allele; BP: base pair location; CHR: chromosome; EAF: effect allele frequency; SE: standard error; SNP: single nucleotide polymorphism.

### The causal effects of RBD on cardiovascular diseases

**Fig 2:**
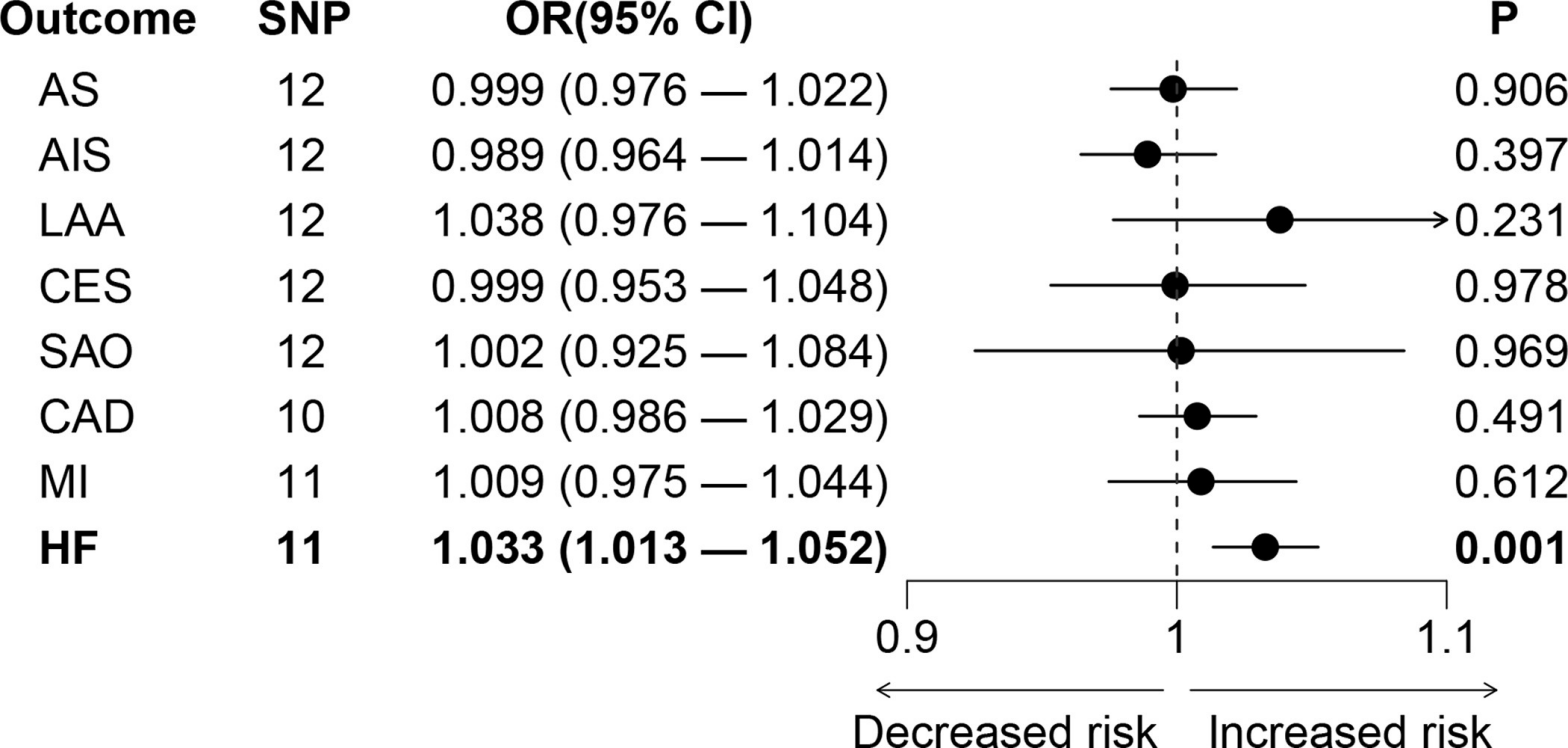
The causal effects of genetically predicted RBD on the risk of cardiovascular diseases. AIS: any ischemic stroke; AS: any stroke; CAD: coronary artery disease; CES: cardioembolic stroke; CI: confidence interval; HF: heart failure; LAA: large artery atherosclerosis stroke; MI: myocardial infarction; OR: odds ratio; RBD: rapid eye movement sleep behavior disorder; SAO: small artery occlusion stroke; SNP: single nucleotide polymorphism.

For stroke, genetically predicted RBD was not associated with AS (OR = 0.999, 95% CI 0.976–1.022, *p* = 0.906) or AIS (OR = 0.989, 95% CI 0.964–1.014, *p* = 0.397; **Fig 2**). Moreover, genetically predicted RBD was not associated with the subtypes of IS (LAA: OR = 1.038, 95% CI 0.976–1.104, *p* = 0.231; CES: OR = 0.999, 95% CI 0.953–1.048, *p* = 0.978; SAO: OR = 1.002, 95% CI 0.925–1.084. *p* = 0.969), either. Leave-one-out analysis suggested that the estimated effects were not significantly changed when excluding any single SNP (**S1 Fig**).

**Fig 3:**
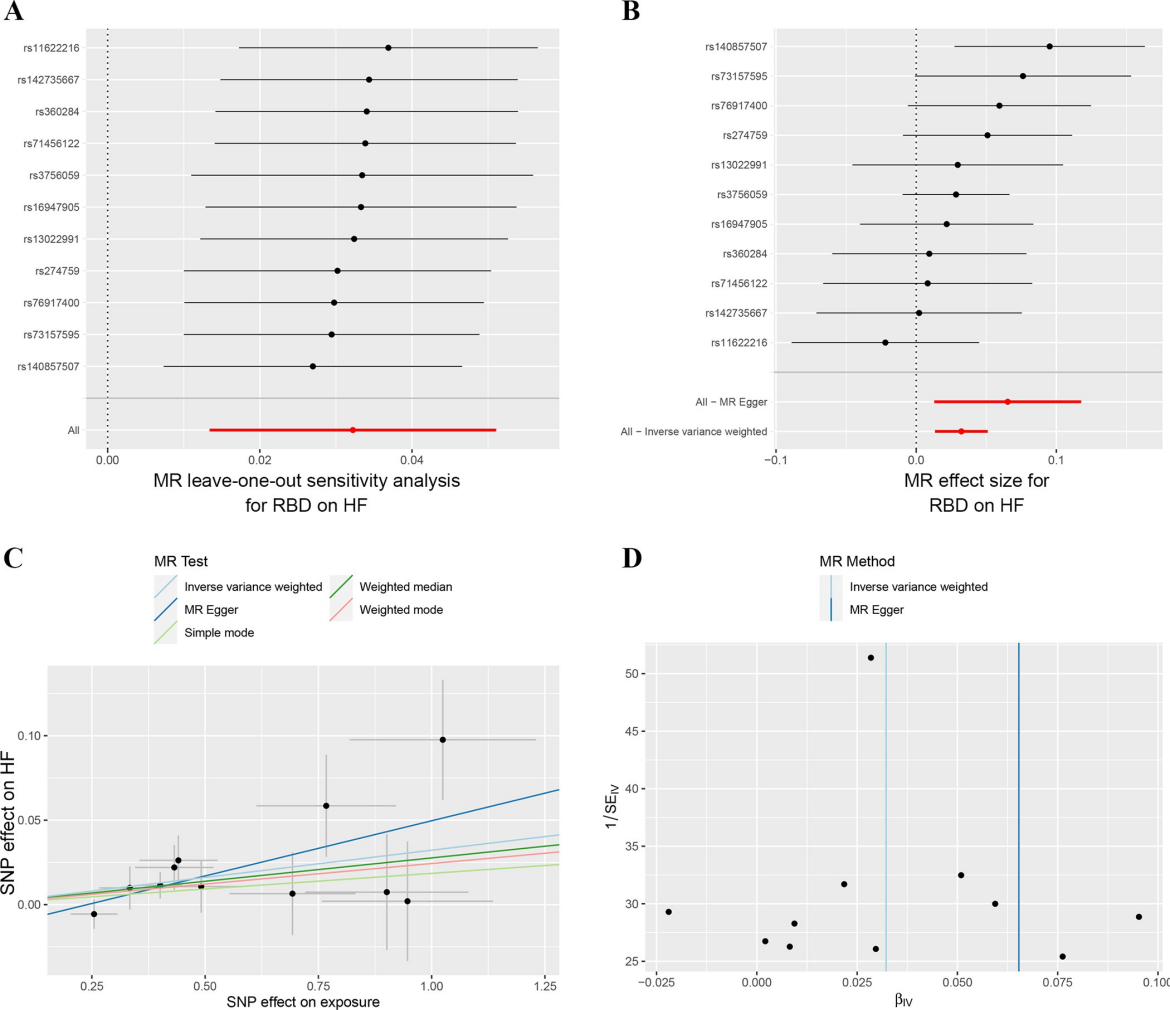
Sensitivity analyses of the causal effects of genetically predicted RBD on the risk of HF. **A.** Leave-one-out analysis; **B.** Forest plot; **C.** Scatter plot; **D.** Funnel plot. HF: heart failure; MR: Mendelian randomization; RBD: rapid eye movement sleep behavior disorder; SNP: single nucleotide polymorphism.

In addition to stroke, genetically predicted RBD was not associated with the risk of CAD (OR = 1.008, 95% CI 0.986–1.029, *p* = 0.491) or MI (OR = 1.009, 95% CI 0.975–1.044, *p* = 0.612; **Fig 2**). While we found a significant causal association of RBD with HF (OR = 1.033, 95% CI 1.013–1.052, *p* = 0.001) after Bonferroni correction (**Fig 2**). The sensitivity analysis of RBD and HF was shown in **Table 2** and **Fig 3**. There was no heterogeneity (*p* = 0.537) or pleiotropy (*p* = 0.218) in the MR analysis between RBD and HF. The statistical power of RBD on cardiovascular diseases was shown in **Table 2**. The power was 0.99 for the association between RBD and HF. Leave-one-out analysis suggested that the result was robust when leaving any single SNP (**Fig 3A**). The complete results of MR analysis including weighted median, weighted mode, simple mode, and MR Egger methods were shown in **S2 Table**.

**Table 2:** Sensitivity analysis and power calculation for Mendelian randomization of rapid eye movement sleep behavior disorder.

Outcome	Heterogeneity		Pleiotropy			Power
	Cochran Q	P	Intercept	Standard error	P	
AS	9.115	0.521	-0.024	0.015	0.138	0.05
AIS	6.521	0.770	-0.037	0.016	0.044	0.24
LAA	7.885	0.640	-0.054	0.040	0.208	0.35
CES	6.893	0.736	-0.104	0.045	0.044	0.05
SAO	13.569	0.194	-0.057	0.031	0.098	0.05
CAD	10.380	0.239	-0.020	0.013	0.165	0.34
MI	18.129	0.034	-0.011	0.023	0.644	0.09
HF	7.974	0.537	-0.016	0.012	0.218	0.99

AIS: any ischemic stroke; AS: any stroke; CAD: coronary artery disease; CES: cardioembolic stroke; HF: heart failure; LAA: large artery atherosclerosis stroke; MI: myocardial infarction; SAO: small artery occlusion.

### The causal effects of cardiovascular diseases on RBD

For instrumental variables selection, there were 7,9,4,4,73,30,12 instrumental variables for AS, AIS, LAA, CES, CAD, MI, and HF identified, respectively (**S3 Table**). And their estimated effects on RBD were shown in **S3 Table**. Regarding SAO, there were no SNPs associated with SAO at genome-wide significance (*p* < 5 × 10^−8^).

**Fig 4:**
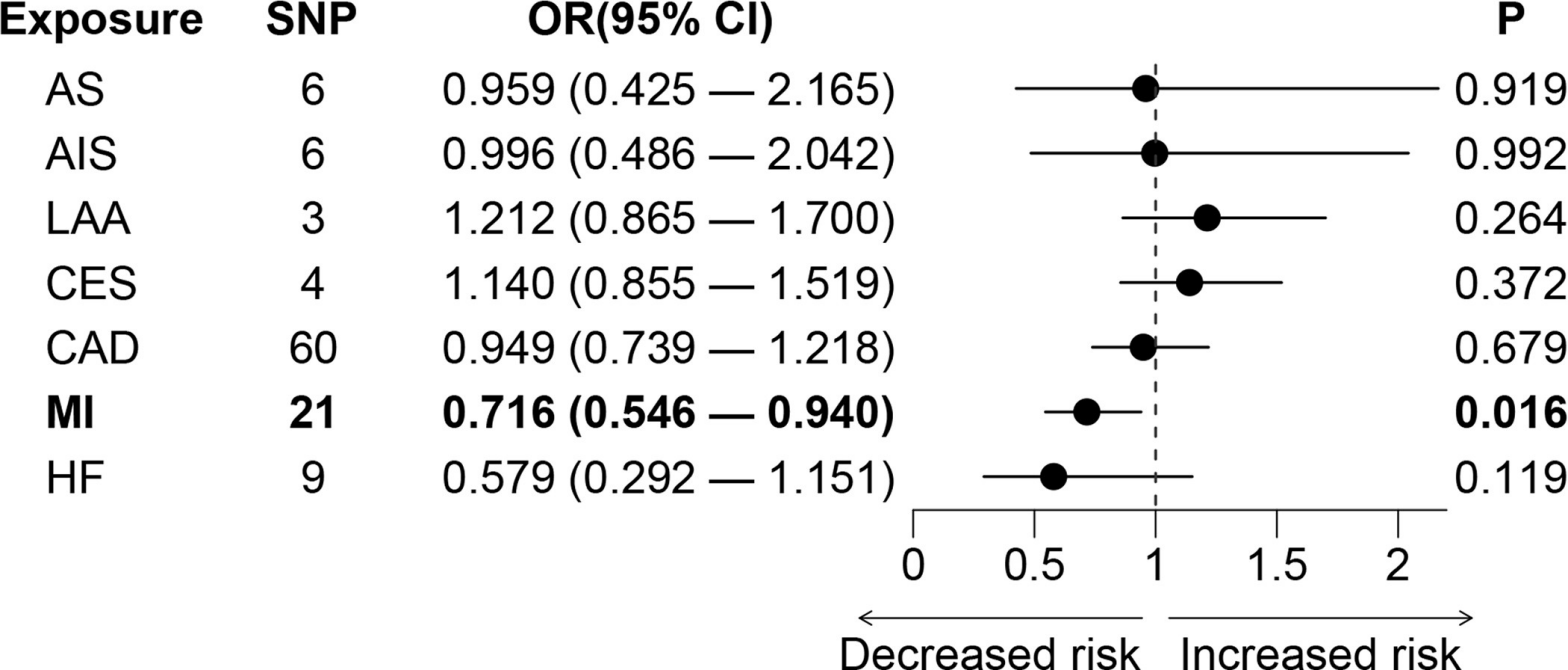
The causal effects of genetically predicted cardiovascular diseases on the risk of RBD. AIS: any ischemic stroke; AS: any stroke; CAD: coronary artery disease; CES: cardioembolic stroke; CI: confidence interval; HF: heart failure; LAA: large artery atherosclerosis stroke; MI: myocardial infarction; OR: odds ratio; RBD: rapid eye movement sleep behavior disorder; SNP: single nucleotide polymorphism.

For stroke, genetically predicted stroke and the subtypes were not associated with the risk of RBD (AS: OR = 0.959, 95% CI 0.425–2.165, *p* = 0.919; AIS: OR = 0.996, 95% CI 0.486–2.042, *p* = 0.992; LAA: OR = 1.212, 95% CI 0.865–1.700, *p* = 0.264; CES: OR = 1.140, 95% CI 0.855–1.519, *p* = 0.372; **Fig 4**). Leave-one-out analysis suggested that the estimated effects were not significantly changed when excluding any single SNP (**S2 Fig**).

In addition to stroke, we found that genetically predicted MI was associated with a lower risk of RBD (OR = 0.716, 95% CI 0.546–0.940, *p* = 0.016; **Fig 4**). While the association was not significant after multiple comparisons. Sensitivity analysis indicated no heterogeneity (*p* = 0.193) or pleiotropy (*p* = 0.361) in the MR analysis between MI and RBD (**Table 3**). In addition, we failed to identify the causal associations between CAD (OR = 0.949, 95% CI 0.739–1.218, *p* = 0.679) or HF (OR = 0.579, 95% CI 0.292–1.151, *p* = 0.119) and RBD (**Fig 4**). The statistical power of cardiovascular diseases on RBD was shown in **Table 3**. The power was 0.85 for the association between MI and RBD. Leave-one-out analysis suggested that the estimated effects were not significantly changed when excluding any single SNP (**S2 Fig**).

**Table 3:** Sensitivity analysis and power calculation for Mendelian randomization of cardiovascular diseases and rapid eye movement sleep behavior disorder.

Exposure	Heterogeneity		Pleiotropy			Power
	Cochran Q	P	Intercept	Standard error	P	
AS	5.092	0.278	-0.235	0.201	0.307	0.05
AIS	2.925	0.570	-0.406	0.208	0.123	0.05
LAA	0.012	0.912	-0.082	0.097	0.552	0.31
CES	0.326	0.849	0.098	0.069	0.293	0.19
CAD	74.617	0.070	0.045	0.019	0.019	/
MI	24.072	0.193	0.030	0.032	0.361	0.85
HF	8.329	0.305	0.064	0.067	0.372	/

AIS: any ischemic stroke; AS: any stroke; CAD: coronary artery disease; CES: cardioembolic stroke; HF: heart failure; LAA: large artery atherosclerosis stroke; MI: myocardial infarction.

/: The effect allele frequency was not available from the GWAS data so the power could not be estimated.

The complete results of MR analysis including weighted median, weighted mode, simple mode, and MR Egger methods were shown in **S4 Table**. And the complete results for sensitivity analysis were shown in **Table 3**.

## Discussion

The current bidirectional MR analysis demonstrated the associations between RBD and cardiovascular diseases at genetic level. We found causal associations between genetically predicted RBD and the risk of HF and the suggestive association between genetically predicted MI and the risk of RBD. However, our analysis failed to identify any causal associations between RBD and stroke and its subtypes.

RBD is a common sleep disorder estimated to occur in about 10% of older community residents [28]. The associations between RBD and neurodegenerative disorders, particularly the synucleinopathies, were frequently reported [29]. However, few studies have investigated the associations between RBD and cerebrovascular diseases such as stroke. A community-based study in China has demonstrated that probable RBD would increase the risk of stroke by nearly 50% [12]. They found that RBD was associated with the risk of both ischemic and hemorrhagic stroke, even after adjusting for other confounding factors [12]. In a single-center study with 2,024 acute IS patients, Tang et al. reported that 10.9% IS patients developed RBD during a three-month follow-up [30]. Intriguingly, RBD patients tended to have smaller infarction volumes, and infarction within brain stem was an independent predictor of RBD [30]. However, our MR analysis failed to identify the causal associations between RBD and stroke. One reason for the established association between RBD and stroke in the previous observational studies is that RBD and stroke shared some common risk factors, including ischemic heart disease and smoking [31]. Therefore, the associations between RBD and stroke need further investigations.

In addition to stroke, our MR analysis highlighted the causal effects of RBD on HF, a common and severe cardiovascular disease [32]. Patients with RBD usually had worse autonomic function, including gastrointestinal and cardiovascular symptoms [33]. Sleep disorders are common in HF patients and nearly 61% patients with HF had central or obstructive sleep apnea [34, 35]. By analyzing the percentage of REM sleep and total time of REM sleep of 4,490 individuals in the Sleep Heart Health Study, Zhao et al. found that REM sleep was independently associated with a lower risk of incident HF [36]. Reduction of REM sleep would induce the dysfunction of neuroendocrine system, including the dysregulation of hypothalamus and pituitary gland and reduction of insulin sensitivity, which could also increase the risk of HF [36, 37]. In addition, previous studies reported alteration of heart rate variability, reduction of heart rate response, and cardiac autonomic dysfunction in patients with RBD [38–40]. However, the association between RBD and HF was rarely reported. RBD was associated with higher risk of diabetes and dyslipidemia, which were known risk factors for HF [41]. The association between RBD and HF could be explained by the disturbance of sleep in RBD patients, which further contributed to metabolic syndromes, including insulin resistance, obesity, and diabetes mellitus [42, 43]. Sleep disturbance was also associated with elevation of nighttime as well as daytime blood pressure [44]. In addition, sleep deprivation also altered inflammatory and immune response, particularly NF-κB signaling pathway activation [45], which was also an essential mechanism for HF [46]. Prolonged activation of NF-κB signaling would trigger chronic inflammation and the production of inflammatory cytokines, thus leading to the damage to cardiac cell and HF [46]. Another explanation for the association between RBD and HR was the impact of α-synuclein. Previous studies have confirmed that α-synuclein was detectable within the heart and cardiovascular autonomic failure was a common symptom in α-synucleinopathies [47, 48]. The α-synuclein in heart may further contributed to myocardial disorders, including cardiomyopathy, septal fibrosis, and ventricular hypertrophy [49]. However, the underlying mechanisms that RBD contributed to HF need further exploration.

The association between RBD and cardiovascular diseases remained unclear due to the influence of confounding factors, which added layers of complexity in identifying the causal association between them. Mendelian randomization analysis was able to bypass the inherent confounding factors and biases of reverse causation present in observational studies, and also overcome the high cost and practical limitations of randomized controlled trials. Therefore, MR study emerged as a promising tool for providing strong evidence of a causal association between RBD and cardiovascular diseases. However, it was noted that despite our inclusion of several potential confounders, additional unmeasured or confounding factors possibly influenced the associations observed in our study.

In addition to the causal effect of RBD on HF, we also observed a suggestive association between MI and RBD. Previous studies have shown that the association between cardiovascular diseases and sleep disorders was considered as a bidirectional interconnection [50]. An observational study showed that MI probably contributed to the loss of sleep-related vagal activation, which was reflected by increased heart rate variability during the REM sleep [51]. Schiza et al. found significantly elevation in the overall sleep duration, sleep efficiency, slow wave sleep, and REM sleep in patients with acute coronary syndrome [52]. They also demonstrated that the associations were obvious in the acute stage but tended to disappear over time [52]. Therefore, whether MI would lead to RBD or not needs further investigations.

The current MR study has some strengths. First, our bidirectional MR analysis leveraged the latest GWAS datasets with large sample size to increase statistical power. Compared with observational design, MR approach would reduce the influence of confounding factors and reverse causations and provide insights into a relatively reliable causal association. However, some limitations of the present MR study should be noticed. First, our analysis was restricted with individuals of European ancestry. Thus, the results of our analysis needed to be further validated in Asian and African populations. Second, in the MR analysis between stroke and RBD, the number of instrumental variables for stroke and its subtypes were limited, which may decrease the statistical power. Therefore, more genetic risk loci associated with stroke and the subtypes needed to be explored. Finally, population stratification, like gender and age, also has an impact on the MR analysis. It should be noted that male sex was an essential risk factor for idiopathic RBD, particularly those aged more than 50 years old [53]. While the RBD GWAS has considered age and gender as covariates in the association model, thus the impact of population stratification was partially controlled [16]. Another limitation of our study was the differences in genetics characteristics of different ethnic groups [54]. For instance, previous studies indicated that some stroke genetic loci displayed population-specific associations [17]. Finally, our study only reported the genetic association between RBD and cardiovascular diseases. The underlying pathogenesis of the association should be further revealed in future studies.

## Conclusion

In conclusion, based on bidirectional MR analysis, we found that genetically predicted RBD was associated with a higher risk of HF. In addition, we found suggestive evidence that MI probably decrease the risk of RBD. However, no causal associations were identified between RBD and stroke and its subtypes. These results suggested the potential associations between RBD and cardiovascular diseases while further studies are needed to reveal the mechanisms of the associations.

## Supporting information

S1 Fig Leave-one-out analyses of the causal effects of genetically predicted RBD on the risk of cardiovascular diseases. (TIF)

S2 Fig Leave-one-out analyses of the causal effects of genetically predicted cardiovascular diseases on the risk of RBD. (TIF)

S1 Table Estimated effects of the instrumental variables for rapid eye movement behavior disorder on the outcomes. (DOCX)

S2 Table Mendelian randomization analysis of rapid eye movement sleep behavior disorder and cardiovascular diseases. (DOCX)

S3 Table Instrumental variables for cardiovascular diseases and the estimated effects on rapid eye movement sleep behavior disorder. (DOCX)

S4 Table Mendelian randomization analysis of cardiovascular diseases and rapid eye movement sleep behavior disorder. (DOCX)
