# Supplementary figures and images for: Genetic associations between Rapid Eye Movement (REM) sleep behavior disorder and cardiovascular diseases
Source: PLoS One. 2024 May 21;19(5):e0301112. doi: 10.1371/journal.pone.0301112 (PMC11108173; doi:10.1371/journal.pone.0301112)

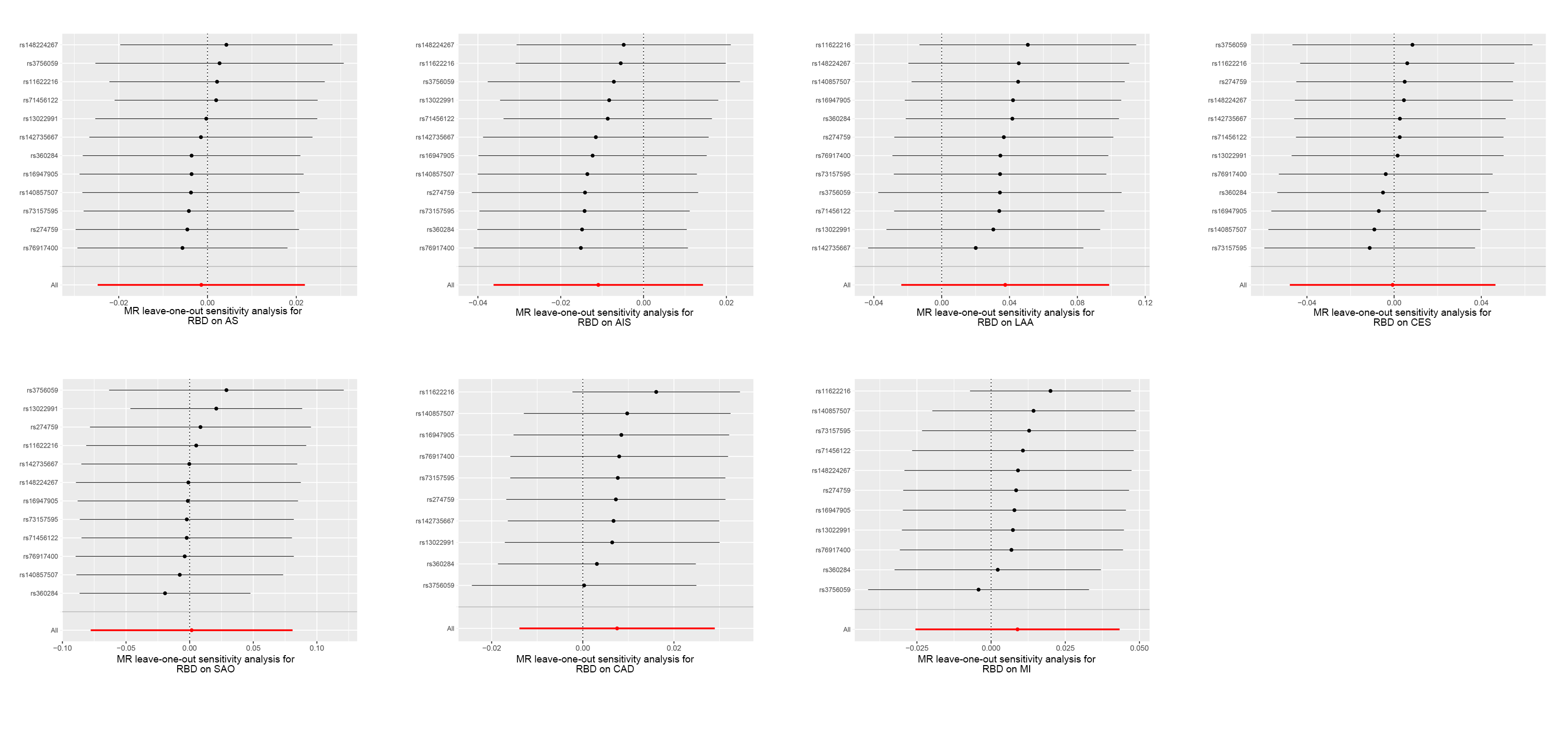

Supplement: S1 Fig — (TIF) [file pone.0301112.s001.tif]

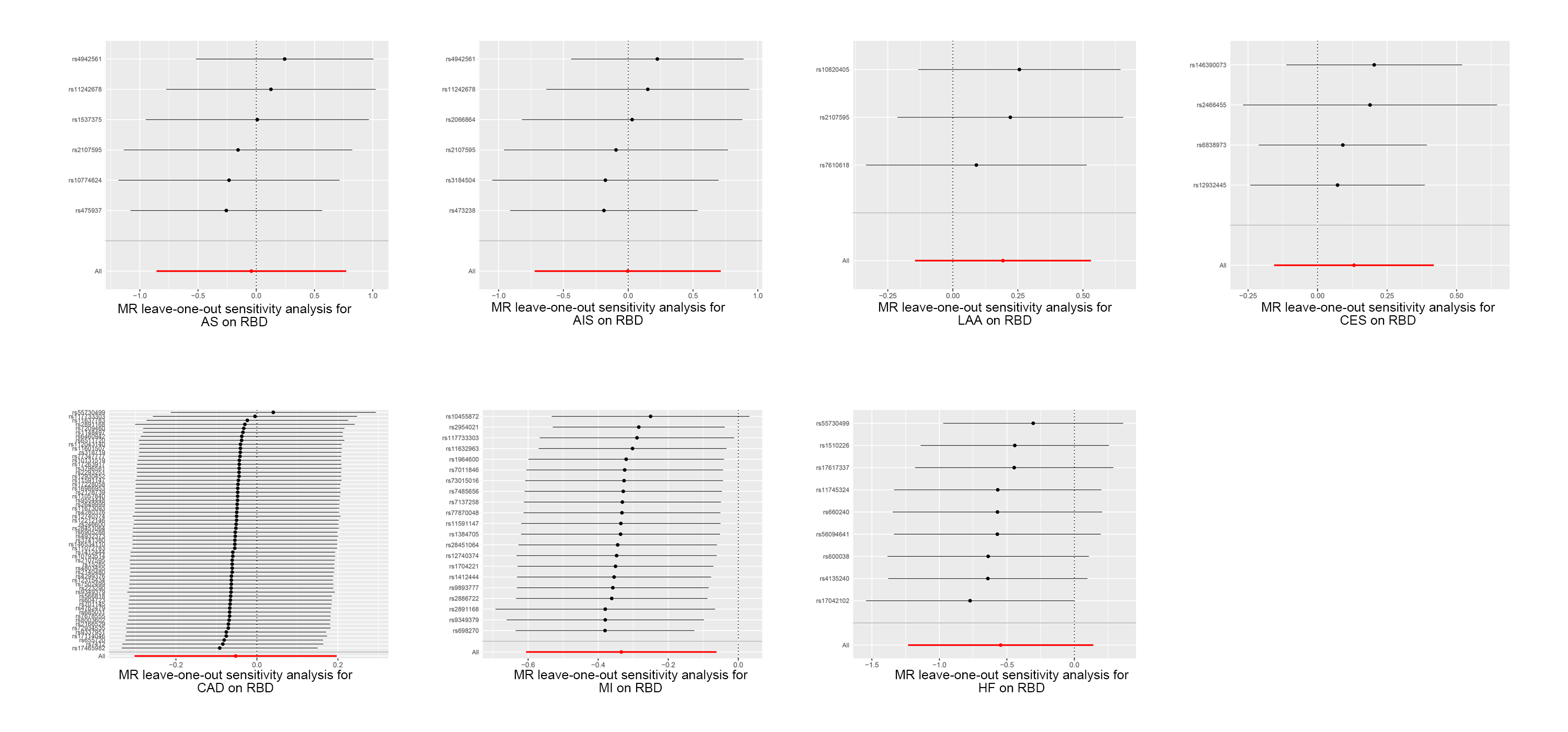

Supplement: S2 Fig — (TIF) [file pone.0301112.s002.tif]
