# Supplementary material for: Genetic associations between Rapid Eye Movement (REM) sleep behavior disorder and cardiovascular diseases
Source: PLoS One. 2024 May 21;19(5):e0301112. doi: 10.1371/journal.pone.0301112 (PMC11108173; doi:10.1371/journal.pone.0301112)
Supplement: S1 Table — (DOCX) [file pone.0301112.s003.docx]

**Supplementary Table 1. Estimated effects of the instrumental variables for rapid eye movement behavior disorder on the outcomes.**

| **SNP** | **BETA**  **_AS** | **SE**  **_AS** | **BETA**  **_AIS** | **SE**  **_AIS** | **BETA**  **_LAA** | **SE_**  **LAA** | **BETA_**  **CES** | **SE_**  **CES** | **BETA_**  **SAO** | **SE_**  **SAO** | **BETA_**  **CAD** | **SE_**  **CAD** | **BETA_**  **MI** | **SE_**  **MI** | **BETA_**  **HF** | **SE_**  **HF** |
| --- | --- | --- | --- | --- | --- | --- | --- | --- | --- | --- | --- | --- | --- | --- | --- | --- |
| rs148224267 | 0.016 | 0.012 | 0.020 | 0.012 | 0.008 | 0.031 | -0.007 | 0.029 | 0.014 | 0.024 | -0.003 | 0.009 | -0.002 | 0.013 | 0.004 | 0.010 |
| rs274759 | -0.011 | 0.015 | -0.007 | 0.017 | -0.019 | 0.043 | 0.025 | 0.040 | 0.022 | 0.033 | -0.004 | 0.012 | -0.006 | 0.018 | -0.022 | 0.013 |
| rs13022991 | -0.007 | 0.016 | -0.019 | 0.018 | 0.053 | 0.045 | -0.116 | 0.043 | -0.014 | 0.035 | 0.007 | 0.011 | 0.010 | 0.016 | 0.010 | 0.013 |
| rs360284 | 0.025 | 0.034 | 0.038 | 0.036 | -0.030 | 0.097 | 0.232 | 0.084 | 0.054 | 0.072 | 0.035 | 0.020 | 0.057 | 0.031 | 0.007 | 0.025 |
| rs3756059 | -0.005 | 0.009 | -0.009 | 0.010 | 0.018 | 0.024 | -0.028 | 0.023 | -0.010 | 0.019 | 0.011 | 0.007 | 0.021 | 0.011 | 0.011 | 0.008 |
| rs140857507 | 0.038 | 0.050 | 0.033 | 0.053 | -0.114 | 0.145 | 0.186 | 0.136 | 0.136 | 0.101 | -0.026 | 0.036 | -0.086 | 0.056 | 0.098 | 0.036 |
| rs76917400 | 0.022 | 0.018 | 0.018 | 0.020 | 0.033 | 0.051 | 0.030 | 0.047 | 0.017 | 0.039 | 0.001 | 0.013 | 0.014 | 0.020 | 0.026 | 0.015 |
| rs71456122 | -0.135 | 0.070 | -0.097 | 0.074 | 0.170 | 0.187 | 0.149 | 0.175 | -0.158 | 0.160 | / | / | -0.014 | 0.044 | 0.007 | 0.034 |
| rs73157595 | 0.044 | 0.041 | 0.045 | 0.045 | 0.080 | 0.115 | 0.072 | 0.113 | 0.161 | 0.085 | 0.004 | 0.026 | -0.037 | 0.039 | 0.059 | 0.030 |
| rs11622216 | -0.011 | 0.011 | -0.019 | 0.011 | -0.029 | 0.028 | -0.010 | 0.026 | -0.020 | 0.022 | -0.021 | 0.008 | -0.032 | 0.012 | -0.006 | 0.009 |
| rs142735667 | 0.000 | 0.048 | -0.001 | 0.050 | 0.263 | 0.114 | 0.040 | 0.135 | -0.055 | 0.097 | 0.021 | 0.038 | / | / | 0.002 | 0.035 |
| rs16947905 | 0.010 | 0.019 | 0.002 | 0.021 | -0.009 | 0.055 | 0.017 | 0.051 | 0.033 | 0.041 | -0.001 | 0.015 | 0.011 | 0.023 | 0.011 | 0.016 |

AIS: any ischemic stroke; AS: any stroke; CAD: coronary artery disease; CES: cardioembolic stroke; HF: heart failure; LAA: large artery atherosclerosis stroke; MI: myocardial infarction; SAO: small artery occlusion; SNP: single nucleotide polymorphism.
