# Supplementary material for: Genetic associations between Rapid Eye Movement (REM) sleep behavior disorder and cardiovascular diseases
Source: PLoS One. 2024 May 21;19(5):e0301112. doi: 10.1371/journal.pone.0301112 (PMC11108173; doi:10.1371/journal.pone.0301112)
Supplement: S2 Table — (DOCX) [file pone.0301112.s004.docx]

**Supplementary Table 2. Mendelian randomization analysis of rapid eye movement sleep behavior disorder and cardiovascular diseases.**

| **Outcome** | **Method** | **SNPs** | **OR (95% CI)** | **P** |
| --- | --- | --- | --- | --- |
| AS | MR Egger | 12 | 1.055 (0.983-1.133) | 0.167 |
| AS | Weighted median | 12 | 0.996 (0.967-1.027) | 0.817 |
| AS | IVW | 12 | 0.999 (0.976-1.022) | 0.906 |
| AS | Simple mode | 12 | 1.030 (0.978-1.085) | 0.281 |
| AS | Weighted mode | 12 | 0.995 (0.955-1.037) | 0.825 |
| AIS | MR Egger | 12 | 1.077 (0.998-1.162) | 0.086 |
| AIS | Weighted median | 12 | 0.993 (0.960-1.028) | 0.706 |
| AIS | IVW | 12 | 0.989 (0.964-1.014) | 0.397 |
| AIS | Simple mode | 12 | 1.024 (0.962-1.090) | 0.468 |
| AIS | Weighted mode | 12 | 0.993 (0.952-1.036) | 0.758 |
| LAA | MR Egger | 12 | 1.176 (0.971-1.425) | 0.128 |
| LAA | Weighted median | 12 | 1.046 (0.965-1.134) | 0.270 |
| LAA | IVW | 12 | 1.038 (0.976-1.104) | 0.231 |
| LAA | Simple mode | 12 | 1.023 (0.897-1.165) | 0.743 |
| LAA | Weighted mode | 12 | 1.032 (0.930-1.145) | 0.563 |
| CES | MR Egger | 12 | 1.139 (0.982-1.320) | 0.116 |
| CES | Weighted median | 12 | 0.970 (0.912-1.032) | 0.330 |
| CES | IVW | 12 | 0.999 (0.953-1.048) | 0.978 |
| CES | Simple mode | 12 | 0.958 (0.864-1.062) | 0.430 |
| CES | Weighted mode | 12 | 0.964 (0.897-1.037) | 0.345 |
| SAO | MR Egger | 12 | 1.278 (1.028-1.589) | 0.052 |
| SAO | Weighted median | 12 | 0.978 (0.906-1.057) | 0.580 |
| SAO | IVW | 12 | 1.002 (0.925-1.084) | 0.969 |
| SAO | Simple mode | 12 | 1.032 (0.911-1.168) | 0.629 |
| SAO | Weighted mode | 12 | 0.975 (0.890-1.069) | 0.603 |
| CAD | MR Egger | 10 | 1.053 (0.992-1.119) | 0.130 |
| CAD | Weighted median | 10 | 1.014 (0.991-1.039) | 0.234 |
| CAD | IVW | 10 | 1.008 (0.986-1.029) | 0.491 |
| CAD | Simple mode | 10 | 1.011 (0.976-1.047) | 0.551 |
| CAD | Weighted mode | 10 | 1.018 (0.988-1.049) | 0.272 |
| MI | MR Egger | 11 | 1.035 (0.927-1.155) | 0.556 |
| MI | Weighted median | 11 | 1.023 (0.987-1.060) | 0.219 |
| MI | IVW | 11 | 1.009 (0.975-1.044) | 0.612 |
| MI | Simple mode | 11 | 1.022 (0.966-1.081) | 0.464 |
| MI | Weighted mode | 11 | 1.029 (0.985-1.075) | 0.232 |
| HF | MR Egger | 11 | 1.067 (1.013-1.125) | 0.037 |
| HF | Weighted median | 11 | 1.028 (1.000-1.057) | 0.050 |
| HF | IVW | 11 | 1.033 (1.013-1.052) | 0.001 |
| HF | Simple mode | 11 | 1.019 (0.975-1.064) | 0.426 |
| HF | Weighted mode | 11 | 1.025 (0.991-1.060) | 0.187 |

AIS: any ischemic stroke; AS: any stroke; CAD: coronary artery disease; CES: cardioembolic stroke; CI: confidence interval; HF: heart failure; IVW: inverse variance weighted; LAA: large artery atherosclerosis stroke; MI: myocardial infarction; MR: Mendelian randomization; OR: odds ratio; SAO: small artery occlusion; SNP: single nucleotide polymorphism.
