# Supplementary material for: Genetic associations between Rapid Eye Movement (REM) sleep behavior disorder and cardiovascular diseases
Source: PLoS One. 2024 May 21;19(5):e0301112. doi: 10.1371/journal.pone.0301112 (PMC11108173; doi:10.1371/journal.pone.0301112)
Supplement: S3 Table — (DOCX) [file pone.0301112.s005.docx]

**Supplementary Table 3. Instrumental variables for cardiovascular diseases and the estimated effects on rapid eye movement sleep behavior disorder.**

| **SNP** | **CHR** | **BP** | **EA** | **OA** | **EAF** | **Exposure** | | | **Outcome** | | |
| --- | --- | --- | --- | --- | --- | --- | --- | --- | --- | --- | --- |
|  |  |  |  |  |  | **BETA** | **SE** | **P** | **BETA** | **SE** | **P** |
| AS |  |  |  |  |  |  |  |  |  |  |  |
| rs2634074 | 4 | 111677041 | A | T | 0.789 | -0.084 | 0.011 | 6.56E-14 | -0.025 | 0.058 | 0.668 |
| rs11242678 | 6 | 1337180 | T | C | 0.255 | 0.064 | 0.011 | 8.71E-10 | -0.061 | 0.058 | 0.290 |
| rs2107595 | 7 | 19049388 | A | G | 0.167 | 0.080 | 0.012 | 3.59E-11 | 0.035 | 0.065 | 0.589 |
| rs1537375 | 9 | 22116071 | C | T | 0.502 | 0.052 | 0.009 | 1.24E-08 | -0.022 | 0.054 | 0.682 |
| rs475937 | 11 | 102687700 | C | A | 0.868 | -0.076 | 0.014 | 2.92E-08 | -0.089 | 0.070 | 0.198 |
| rs10774624 | 12 | 111833788 | A | G | 0.529 | -0.065 | 0.009 | 4.04E-12 | -0.042 | 0.050 | 0.398 |
| rs4942561 | 13 | 47209347 | T | G | 0.758 | 0.064 | 0.011 | 2.05E-09 | -0.096 | 0.056 | 0.088 |
| AIS |  |  |  |  |  |  |  |  |  |  |  |
| rs2758612 | 1 | 156205301 | C | T | 0.355 | -0.065 | 0.011 | 3.68E-09 |  |  |  |
| rs34311906 | 4 | 113732090 | C | T | 0.402 | 0.065 | 0.011 | 1.07E-08 |  |  |  |
| rs2634074 | 4 | 111677041 | A | T | 0.788 | -0.094 | 0.012 | 5.90E-15 | -0.025 | 0.058 | 0.668 |
| rs2066864 | 4 | 155525695 | A | G | 0.245 | 0.063 | 0.012 | 3.51E-08 | -0.016 | 0.057 | 0.782 |
| rs11242678 | 6 | 1337180 | T | C | 0.255 | 0.072 | 0.011 | 2.70E-10 | -0.061 | 0.058 | 0.290 |
| rs2107595 | 7 | 19049388 | A | G | 0.167 | 0.088 | 0.013 | 2.33E-11 | 0.035 | 0.065 | 0.589 |
| rs473238 | 11 | 102700360 | C | T | 0.867 | -0.083 | 0.015 | 1.65E-08 | -0.092 | 0.069 | 0.187 |
| rs3184504 | 12 | 111884608 | C | T | 0.528 | -0.078 | 0.010 | 1.23E-14 | -0.038 | 0.048 | 0.434 |
| rs4942561 | 13 | 47209347 | T | G | 0.759 | 0.066 | 0.012 | 1.77E-08 | -0.096 | 0.056 | 0.088 |
| LAA |  |  |  |  |  |  |  |  |  |  |  |
| rs7610618 | 3 | 149157706 | T | C | 0.013 | 0.845 | 0.149 | 1.44E-08 | 0.314 | 0.242 | 0.194 |
| rs2107595 | 7 | 19049388 | A | G | 0.168 | 0.236 | 0.032 | 1.44E-13 | 0.035 | 0.065 | 0.589 |
| rs10820405 | 9 | 106010237 | A | G | 0.185 | -0.181 | 0.033 | 4.51E-08 | 0.001 | 0.063 | 0.992 |
| rs476762 | 11 | 102710707 | A | T | 0.133 | 0.201 | 0.035 | 1.22E-08 | 0.081 | 0.069 | 0.242 |
| CES |  |  |  |  |  |  |  |  |  |  |  |
| rs146390073 | 1 | 241306248 | T | C | 0.022 | 0.669 | 0.120 | 2.20E-08 | -0.142 | 0.234 | 0.544 |
| rs2466455 | 4 | 111685615 | T | C | 0.783 | -0.299 | 0.022 | 2.75E-41 | -0.026 | 0.058 | 0.659 |
| rs6838973 | 4 | 111765495 | T | C | 0.434 | -0.108 | 0.020 | 3.58E-08 | -0.054 | 0.051 | 0.286 |
| rs12932445 | 16 | 73069888 | C | T | 0.181 | 0.176 | 0.025 | 6.88E-13 | 0.076 | 0.064 | 0.233 |
| CAD |  |  |  |  |  |  |  |  |  |  |  |
| rs11591147 | 1 | 55505647 | T | G | NA | -0.237 | 0.027 | 1.50E-18 | 0.082 | 0.169 | 0.626 |
| rs12740374 | 1 | 109817590 | T | G | NA | -0.104 | 0.008 | 8.20E-36 | 0.012 | 0.058 | 0.839 |
| rs17114046 | 1 | 56966350 | G | A | NA | -0.097 | 0.012 | 2.80E-16 | -0.144 | 0.086 | 0.093 |
| rs17465982 | 1 | 222837939 | A | G | NA | 0.066 | 0.008 | 7.30E-18 | 0.147 | 0.055 | 0.007 |
| rs4299376 | 2 | 44072576 | T | G | NA | -0.045 | 0.008 | 1.50E-09 | -0.046 | 0.050 | 0.355 |
| rs2166529 | 2 | 85742175 | T | G | NA | 0.056 | 0.007 | 1.10E-15 | 0.059 | 0.048 | 0.221 |
| rs16986953 | 2 | 19942473 | A | G | NA | 0.081 | 0.013 | 1.40E-09 | -0.050 | 0.091 | 0.585 |
| rs1250259 | 2 | 216300482 | A | T | NA | -0.047 | 0.008 | 2.90E-09 | 0.066 | 0.056 | 0.238 |
| rs72934535 | 2 | 203968973 | C | T | NA | 0.100 | 0.012 | 3.70E-18 | 0.081 | 0.075 | 0.284 |
| rs4678145 | 3 | 124450081 | C | G | NA | 0.064 | 0.010 | 6.90E-10 | -0.080 | 0.073 | 0.272 |
| rs2293251 | 3 | 138124114 | G | T | NA | 0.061 | 0.010 | 3.40E-10 | -0.056 | 0.067 | 0.401 |
| rs77347777 | 3 | 52848207 | T | C | NA | -0.061 | 0.011 | 3.70E-08 | 0.107 | 0.083 | 0.197 |
| rs701145 | 3 | 154054799 | C | T | NA | 0.066 | 0.010 | 2.40E-11 | 0.086 | 0.071 | 0.228 |
| rs112043140 | 3 | 46585022 | T | C | NA | 0.046 | 0.008 | 2.90E-08 | -0.084 | 0.060 | 0.166 |
| rs655720 | 3 | 136103267 | T | C | NA | 0.054 | 0.008 | 2.90E-12 | 0.126 | 0.054 | 0.019 |
| rs10857147 | 4 | 81181072 | T | A | NA | 0.046 | 0.008 | 2.30E-09 | -0.054 | 0.059 | 0.365 |
| rs3796581 | 4 | 156642884 | G | A | NA | -0.069 | 0.009 | 2.60E-14 | 0.042 | 0.060 | 0.481 |
| rs7678555 | 4 | 120909501 | C | A | NA | 0.046 | 0.008 | 3.70E-09 | 0.073 | 0.052 | 0.161 |
| rs17612693 | 4 | 148365339 | A | T | NA | 0.081 | 0.010 | 3.20E-16 | -0.142 | 0.071 | 0.048 |
| rs246600 | 5 | 142516897 | T | C | NA | 0.044 | 0.007 | 4.00E-10 | -0.008 | 0.048 | 0.868 |
| rs17263917 | 5 | 9552338 | A | G | NA | -0.062 | 0.010 | 3.20E-10 | 0.054 | 0.064 | 0.398 |
| rs685031 | 6 | 31881731 | A | G | NA | 0.046 | 0.007 | 3.00E-10 | 0.073 | 0.052 | 0.162 |
| rs9349379 | 6 | 12903957 | G | A | NA | 0.093 | 0.007 | 6.90E-39 | 0.022 | 0.052 | 0.672 |
| rs6905288 | 6 | 43758873 | A | G | NA | 0.041 | 0.007 | 4.20E-09 | 0.002 | 0.052 | 0.964 |
| rs223290 | 6 | 57106712 | C | T | NA | -0.097 | 0.014 | 6.70E-12 | -0.080 | 0.094 | 0.394 |
| rs1966248 | 6 | 134159622 | T | A | NA | -0.071 | 0.008 | 1.50E-20 | -0.058 | 0.054 | 0.285 |
| rs146534110 | 6 | 160578069 | T | G | NA | 0.209 | 0.031 | 2.20E-11 | 0.026 | 0.220 | 0.904 |
| rs117733303 | 6 | 160922870 | G | A | NA | 0.452 | 0.026 | 8.40E-66 | -0.444 | 0.220 | 0.044 |
| rs55730499 | 6 | 161005610 | T | C | NA | 0.311 | 0.013 | 6.30E-120 | -0.290 | 0.109 | 0.008 |
| rs12212146 | 6 | 161125454 | C | T | NA | -0.089 | 0.014 | 2.10E-10 | 0.029 | 0.104 | 0.781 |
| rs6460942 | 7 | 12420989 | C | T | NA | -0.059 | 0.010 | 7.60E-09 | 0.106 | 0.069 | 0.124 |
| rs3918226 | 7 | 150690176 | T | C | NA | 0.097 | 0.013 | 2.30E-13 |  |  |  |
| rs2107595 | 7 | 19049388 | A | G | NA | 0.077 | 0.010 | 9.40E-16 | 0.035 | 0.065 | 0.589 |
| rs28601761 | 8 | 126500031 | G | C | NA | -0.057 | 0.007 | 2.80E-15 | 0.127 | 0.048 | 0.009 |
| rs1148497 | 8 | 102856517 | G | A | NA | -0.046 | 0.008 | 4.70E-08 | 0.115 | 0.058 | 0.047 |
| rs15285 | 8 | 19824667 | T | C | NA | -0.048 | 0.008 | 4.00E-10 | -0.037 | 0.051 | 0.470 |
| rs2891168 | 9 | 22098619 | G | A | NA | 0.170 | 0.007 | 1.80E-131 | -0.033 | 0.049 | 0.503 |
| rs2140480 | 9 | 110526109 | C | T | NA | -0.044 | 0.008 | 2.20E-08 | -0.045 | 0.053 | 0.396 |
| rs1412444 | 10 | 91002927 | T | C | NA | 0.049 | 0.007 | 1.40E-11 | 0.028 | 0.051 | 0.586 |
| rs9337951 | 10 | 30317073 | A | G | NA | 0.050 | 0.008 | 4.60E-11 | 0.106 | 0.053 | 0.045 |
| rs7896600 | 10 | 12255175 | C | G | NA | 0.045 | 0.008 | 2.30E-08 | 0.021 | 0.055 | 0.706 |
| rs10793514 | 10 | 44496971 | C | T | NA | 0.051 | 0.007 | 4.40E-12 | 0.027 | 0.051 | 0.591 |
| rs566818 | 11 | 75158350 | G | A | NA | -0.042 | 0.008 | 3.20E-08 | -0.069 | 0.054 | 0.201 |
| rs11601507 | 11 | 5701074 | A | C | NA | 0.081 | 0.014 | 2.10E-09 | -0.131 | 0.100 | 0.192 |
| rs604723 | 11 | 100610546 | C | T | NA | 0.050 | 0.008 | 2.80E-10 | 0.063 | 0.055 | 0.248 |
| rs2128739 | 11 | 103673277 | C | A | NA | -0.066 | 0.008 | 1.00E-17 | 0.021 | 0.052 | 0.684 |
| rs3741380 | 11 | 65349063 | A | G | NA | 0.041 | 0.007 | 3.60E-09 | 0.006 | 0.049 | 0.902 |
| rs964184 | 11 | 116648917 | C | G | NA | -0.060 | 0.010 | 2.20E-09 | -0.006 | 0.069 | 0.930 |
| rs2649999 | 12 | 121380544 | C | T | NA | -0.043 | 0.008 | 1.60E-08 | 0.022 | 0.052 | 0.667 |
| rs12315434 | 12 | 57780936 | C | A | NA | -0.050 | 0.008 | 2.80E-09 | -0.066 | 0.062 | 0.287 |
| rs4766578 | 12 | 111904371 | A | T | NA | -0.065 | 0.007 | 5.30E-20 | -0.030 | 0.048 | 0.531 |
| rs10841443 | 12 | 20220033 | G | C | NA | 0.043 | 0.007 | 5.20E-09 | 0.081 | 0.052 | 0.123 |
| rs4762479 | 12 | 95495041 | T | C | NA | -0.085 | 0.013 | 2.50E-10 | -0.121 | 0.093 | 0.196 |
| rs11057840 | 12 | 125316055 | C | A | NA | 0.071 | 0.010 | 1.40E-12 | -0.031 | 0.068 | 0.654 |
| rs11617955 | 13 | 110818102 | A | T | NA | -0.085 | 0.011 | 4.00E-14 | 0.033 | 0.074 | 0.658 |
| rs11619113 | 13 | 110918660 | G | C | NA | 0.058 | 0.010 | 3.00E-08 | 0.103 | 0.069 | 0.132 |
| rs9554448 | 13 | 98859722 | C | T | NA | -0.063 | 0.011 | 3.90E-08 | 0.047 | 0.083 | 0.572 |
| rs8003602 | 14 | 100148961 | C | T | NA | 0.055 | 0.008 | 4.10E-12 | 0.078 | 0.057 | 0.174 |
| rs10131519 | 14 | 100359294 | C | T | NA | 0.050 | 0.009 | 2.60E-08 | -0.068 | 0.064 | 0.293 |
| rs17228058 | 15 | 67450305 | G | A | NA | -0.052 | 0.008 | 4.00E-10 | 0.034 | 0.057 | 0.554 |
| rs11637783 | 15 | 79139000 | C | T | NA | -0.064 | 0.007 | 9.70E-20 | 0.093 | 0.048 | 0.056 |
| rs4932373 | 15 | 91429287 | C | A | NA | 0.069 | 0.007 | 3.30E-20 | 0.000 | 0.054 | 0.995 |
| rs11072783 | 15 | 78965966 | A | G | NA | -0.056 | 0.009 | 1.20E-09 | -0.010 | 0.064 | 0.878 |
| rs12930452 | 16 | 75462055 | G | A | NA | 0.049 | 0.007 | 5.50E-12 | -0.038 | 0.049 | 0.439 |
| rs7209460 | 17 | 2048713 | T | C | NA | 0.052 | 0.008 | 7.60E-12 | -0.093 | 0.053 | 0.076 |
| rs7502499 | 17 | 47490102 | A | G | NA | 0.053 | 0.008 | 3.30E-12 | 0.041 | 0.051 | 0.418 |
| rs6511720 | 19 | 11202306 | T | G | NA | -0.108 | 0.011 | 3.90E-24 | 0.068 | 0.074 | 0.357 |
| rs11673093 | 19 | 45742094 | A | G | NA | 0.044 | 0.008 | 3.00E-08 | -0.025 | 0.057 | 0.660 |
| rs318719 | 19 | 11496981 | C | T | NA | 0.075 | 0.013 | 5.60E-09 | -0.139 | 0.102 | 0.172 |
| rs4280376 | 19 | 17861209 | C | T | NA | 0.056 | 0.009 | 5.80E-10 | -0.021 | 0.066 | 0.745 |
| rs4803455 | 19 | 41851509 | A | C | NA | -0.050 | 0.007 | 1.10E-12 | -0.031 | 0.048 | 0.524 |
| rs7412 | 19 | 45412079 | T | C | NA | -0.137 | 0.013 | 7.30E-27 | -0.193 | 0.102 | 0.058 |
| rs28451064 | 21 | 35593827 | A | G | NA | 0.099 | 0.011 | 2.70E-20 | -0.008 | 0.072 | 0.909 |
| MI |  |  |  |  |  |  |  |  |  |  |  |
| rs11591147 | 1 | 55505647 | T | G | 0.017 | -0.271 | 0.042 | 8.10E-11 | 0.082 | 0.169 | 0.626 |
| rs4846384 | 1 | 222797614 | G | C | 0.691 | 0.093 | 0.012 | 3.10E-15 | 0.122 | 0.055 | 0.026 |
| rs12740374 | 1 | 109817590 | T | G | 0.222 | -0.111 | 0.013 | 5.70E-18 | 0.012 | 0.058 | 0.839 |
| rs2886722 | 2 | 85742297 | G | A | 0.404 | 0.067 | 0.011 | 1.10E-09 | 0.040 | 0.049 | 0.410 |
| rs698270 | 3 | 136109512 | G | A | 0.768 | 0.079 | 0.013 | 4.80E-10 | 0.096 | 0.057 | 0.093 |
| rs117733303 | 6 | 160922870 | G | A | 0.018 | 0.457 | 0.040 | 3.90E-30 | -0.444 | 0.220 | 0.044 |
| rs1966248 | 6 | 134159622 | T | A | 0.309 | -0.089 | 0.012 | 3.20E-14 | -0.058 | 0.054 | 0.285 |
| rs10455872 | 6 | 161010118 | G | A | 0.075 | 0.328 | 0.020 | 4.00E-58 | -0.282 | 0.109 | 0.010 |
| rs9349379 | 6 | 12903957 | G | A | 0.402 | 0.120 | 0.011 | 1.40E-27 | 0.022 | 0.052 | 0.672 |
| rs3918226 | 7 | 150690176 | T | C | 0.078 | 0.120 | 0.020 | 3.80E-09 |  |  |  |
| rs7011846 | 8 | 19785656 | A | G | 0.032 | 0.201 | 0.032 | 3.30E-10 | -0.149 | 0.163 | 0.361 |
| rs2954021 | 8 | 126482077 | G | A | 0.507 | -0.062 | 0.011 | 8.90E-09 | 0.138 | 0.048 | 0.004 |
| rs376993806 | 9 | 139246588 | G | A | 0.717 | 0.069 | 0.012 | 1.70E-08 |  |  |  |
| rs2891168 | 9 | 22098619 | G | A | 0.483 | 0.188 | 0.011 | 2.00E-68 | -0.033 | 0.049 | 0.503 |
| rs1412444 | 10 | 91002927 | T | C | 0.344 | 0.067 | 0.011 | 2.30E-09 | 0.028 | 0.051 | 0.586 |
| rs1704221 | 10 | 44710930 | A | G | 0.107 | -0.109 | 0.017 | 3.60E-10 | -0.023 | 0.079 | 0.767 |
| rs1964600 | 11 | 1680664 | A | G | 0.248 | 0.069 | 0.012 | 3.20E-08 | -0.063 | 0.056 | 0.257 |
| rs1384705 | 11 | 103696851 | T | C | 0.706 | -0.078 | 0.012 | 3.20E-11 | 0.022 | 0.051 | 0.672 |
| rs7137258 | 12 | 54512164 | A | C | 0.062 | 0.126 | 0.023 | 3.80E-08 | -0.062 | 0.113 | 0.587 |
| rs10841443 | 12 | 20220033 | G | C | 0.659 | 0.065 | 0.011 | 1.80E-08 | 0.081 | 0.052 | 0.123 |
| rs7485656 | 12 | 125315647 | G | A | 0.151 | 0.084 | 0.015 | 3.00E-08 | -0.048 | 0.068 | 0.482 |
| rs11617955 | 13 | 110818102 | A | T | 0.111 | -0.104 | 0.017 | 2.20E-09 | 0.033 | 0.074 | 0.658 |
| rs11632963 | 15 | 79132644 | A | G | 0.505 | 0.064 | 0.011 | 2.50E-09 | -0.098 | 0.049 | 0.046 |
| rs12906125 | 15 | 91427612 | A | G | 0.321 | 0.085 | 0.012 | 1.30E-13 |  |  |  |
| rs77870048 | 16 | 69965021 | T | C | 0.050 | -0.138 | 0.025 | 1.90E-08 | 0.063 | 0.140 | 0.650 |
| rs9893777 | 17 | 47336686 | T | G | 0.465 | -0.062 | 0.011 | 1.10E-08 | -0.038 | 0.048 | 0.434 |
| rs10404176 | 19 | 17829393 | G | A | 0.539 | 0.060 | 0.011 | 2.40E-08 |  |  |  |
| rs73015016 | 19 | 11191300 | A | G | 0.123 | -0.105 | 0.016 | 1.70E-10 | 0.061 | 0.074 | 0.409 |
| rs1065853 | 19 | 45413233 | T | G | 0.080 | -0.163 | 0.020 | 1.90E-16 |  |  |  |
| rs28451064 | 21 | 35593827 | A | G | 0.127 | 0.113 | 0.017 | 6.80E-12 | -0.008 | 0.072 | 0.909 |
| HF |  |  |  |  |  |  |  |  |  |  |  |
| rs660240 | 1 | 109817838 | C | T | NA | 0.061 | 0.010 | 3.25E-10 | -0.022 | 0.059 | 0.717 |
| rs17042102 | 4 | 111668626 | A | G | NA | 0.110 | 0.012 | 5.71E-20 | 0.018 | 0.072 | 0.804 |
| rs11745324 | 5 | 137012171 | A | G | NA | -0.053 | 0.010 | 2.34E-08 | 0.017 | 0.056 | 0.755 |
| rs1510226 | 6 | 160816409 | C | T | NA | 0.162 | 0.029 | 1.27E-08 | -0.325 | 0.202 | 0.108 |
| rs55730499 | 6 | 161005610 | T | C | NA | 0.106 | 0.016 | 1.83E-11 | -0.290 | 0.109 | 0.008 |
| rs4135240 | 6 | 36647680 | C | T | NA | -0.049 | 0.008 | 6.84E-09 | -0.016 | 0.050 | 0.743 |
| rs600038 | 9 | 136151806 | C | T | NA | 0.057 | 0.010 | 3.68E-09 | 0.014 | 0.057 | 0.805 |
| rs1556516 | 9 | 22100176 | C | G | NA | 0.062 | 0.008 | 1.57E-15 | -0.038 | 0.049 | 0.438 |
| rs4746140 | 10 | 75417249 | C | G | NA | -0.067 | 0.011 | 1.10E-09 | -0.007 | 0.068 | 0.917 |
| rs17617337 | 10 | 121426884 | T | C | NA | -0.056 | 0.010 | 3.65E-09 | 0.083 | 0.058 | 0.154 |
| rs4766578 | 12 | 111904371 | A | T | NA | -0.043 | 0.008 | 4.90E-08 | -0.030 | 0.048 | 0.531 |
| rs56094641 | 16 | 53806453 | G | A | NA | 0.045 | 0.008 | 1.21E-08 | -0.013 | 0.049 | 0.787 |

A1: effect allele; A2: other allele; AIS: any ischemic stroke; AS: any stroke; BP: base pair location; CAD: coronary artery disease; CES: cardioembolic stroke; CHR: chromosome; EAF: effect allele frequency; HF: heart failure; LAA: large artery atherosclerosis stroke; MI: myocardial infarction; SAO: small artery occlusion; SE: standard error; SNP: single nucleotide polymorphism.
