# Supplementary material for: Genetic associations between Rapid Eye Movement (REM) sleep behavior disorder and cardiovascular diseases
Source: PLoS One. 2024 May 21;19(5):e0301112. doi: 10.1371/journal.pone.0301112 (PMC11108173; doi:10.1371/journal.pone.0301112)
Supplement: S4 Table — (DOCX) [file pone.0301112.s006.docx]

**Supplementary Table 4. Mendelian randomization analysis of cardiovascular diseases and rapid eye movement sleep behavior disorder.**

| **Outcome** | **Method** | **SNPs** | **OR (95% CI)** | **P** |
| --- | --- | --- | --- | --- |
| AS | Weighted median | 6 | 1.302 (0.506-3.347) | 0.584 |
| AS | IVW | 6 | 0.959 (0.425-2.165) | 0.919 |
| AS | Simple mode | 6 | 1.656 (0.291-9.430) | 0.595 |
| AS | Weighted mode | 6 | 1.720 (0.389-7.607) | 0.506 |
| AIS | Weighted median | 6 | 1.480 (0.624-3.508) | 0.373 |
| AIS | IVW | 6 | 0.996 (0.486-2.042) | 0.992 |
| AIS | Simple mode | 6 | 1.392 (0.358-5.419) | 0.653 |
| AIS | Weighted mode | 6 | 1.496 (0.478-4.681) | 0.520 |
| LAA | MR Egger | 3 | 1.610 (0.770-3.363) | 0.426 |
| LAA | Weighted median | 3 | 1.197 (0.788-1.816) | 0.399 |
| LAA | IVW | 3 | 1.212 (0.865-1.700) | 0.264 |
| LAA | Simple mode | 3 | 1.111 (0.671-1.840) | 0.721 |
| LAA | Weighted mode | 3 | 1.162 (0.711-1.899) | 0.610 |
| CES | MR Egger | 4 | 0.773 (0.420-1.422) | 0.495 |
| CES | Weighted median | 4 | 1.133 (0.810-1.583) | 0.466 |
| CES | IVW | 4 | 1.140 (0.855-1.519) | 0.372 |
| CES | Simple mode | 4 | 1.504 (0.913-2.479) | 0.207 |
| CES | Weighted mode | 4 | 1.092 (0.753-1.585) | 0.674 |
| CAD | MR Egger | 60 | 0.554 (0.336-0.913) | 0.024 |
| CAD | Weighted median | 60 | 0.826 (0.560-1.219) | 0.336 |
| CAD | IVW | 60 | 0.949 (0.739-1.218) | 0.679 |
| CAD | Simple mode | 60 | 0.700 (0.299-1.639) | 0.414 |
| CAD | Weighted mode | 60 | 0.721 (0.454-1.145) | 0.171 |
| MI | MR Egger | 21 | 0.565 (0.321-0.995) | 0.063 |
| MI | Weighted median | 21 | 0.795 (0.545-1.160) | 0.234 |
| MI | IVW | 21 | 0.716 (0.546-0.940) | 0.016 |
| MI | Simple mode | 21 | 0.664 (0.330-1.337) | 0.265 |
| MI | Weighted mode | 21 | 0.799 (0.498-1.283) | 0.365 |
| HF | MR Egger | 9 | 0.226 (0.029-1.767) | 0.199 |
| HF | Weighted median | 9 | 0.749 (0.309-1.819) | 0.524 |
| HF | IVW | 9 | 0.579 (0.292-1.151) | 0.119 |
| HF | Simple mode | 9 | 0.944 (0.260-3.430) | 0.933 |
| HF | Weighted mode | 9 | 1.026 (0.345-3.054) | 0.964 |

AIS: any ischemic stroke; AS: any stroke; CAD: coronary artery disease; CES: cardioembolic stroke; CI: confidence interval; HF: heart failure; IVW: inverse variance weighted; LAA: large artery atherosclerosis stroke; MI: myocardial infarction; MR: Mendelian randomization; OR: odds ratio; SAO: small artery occlusion; SNP: single nucleotide polymorphism.
